# Organizational aspects and implementation of data systems in large-scale epidemiological studies in less developed countries

**DOI:** 10.1186/1471-2458-6-86

**Published:** 2006-04-04

**Authors:** Mohammad Ali, Jin-Kyung Park, Lorenz von Seidlein, Camilo J Acosta, Jacqueline L Deen, John D Clemens

**Affiliations:** 1 International Vaccine Institute, SNU Research Park, San 4-8 Bongcheon-7 dong, Kwanak-gu, Seoul, Korea

## Abstract

**Background:**

In the conduct of epidemiological studies in less developed countries, while great emphasis is placed on study design, data collection, and analysis, often little attention is paid to data management. As a consequence, investigators working in these countries frequently face challenges in cleaning, analyzing and interpreting data. In most research settings, the data management team is formed with temporary and unskilled persons. A proper working environment and training or guidance in constructing a reliable database is rarely available. There is little information available that describes data management problems and solutions to those problems. Usually a line or two can be obtained in the methods section of research papers stating that the data are doubly-entered and that outliers and inconsistencies were removed from the data. Such information provides little assurance that the data are reliable. There are several issues in data management that if not properly practiced may create an unreliable database, and outcomes of this database will be spurious.

**Results:**

We have outlined the data management practices for epidemiological studies that we have modeled for our research sites in seven Asian countries and one African country.

**Conclusion:**

Information from this model data management structure may help others construct reliable databases for large-scale epidemiological studies in less developed countries.

## Background

Large scale epidemiological studies are being conducted with increasing frequency in less developed countries (LDCs). While great attention is placed on sample size estimation, statistical analysis, and primary data collection (in the field and in the laboratory), surprisingly little attention is paid to the computerization of the data [1-3]. As a result, an efficient data management system is often not available in epidemiological studies in these countries. Literature describing practical problems in data management is scarce [4,5]. Despite enormous advances in information science technology over the last two decades, data management practices in studies in LDC are usually less than ideal – a fundamental requirement that investigators frequently overlook. In most instances, individuals working in data management are not familiar with concepts about epidemiological studies, specific objectives of the studies, and the complexities in management of epidemiological study data; thus impinging on the quality and reliability of the database.

The success of an epidemiological study depends on many factors including reliable database support. In many studies, a low rate of errors such as outliers and inconsistencies is used to indicate reliability of the database. A low rate of errors does not guarantee that the database is reliable. Even a single error can be a sign of systems malfunction. The data management systems should ensure accurate and complete data collection; efficient design of the database; easy data entry through the use of data collection forms; security of the databases; structured methods for detecting and correcting keypunching errors, implausible values, false zeros, and inconsistencies; and integrity of the data sets and the protection of privacy. The management systems should also include methods of organizing, sorting, linking, grouping, and selecting data for analysis.

In the context of the Diseases of the Most Impoverished (DOMI) program undertaken by the International Vaccine Institute (IVI), Seoul, Korea [6], we had the opportunity to design and maintain computerized data management systems for several large-scale epidemiological studies including disease surveillance and vaccine effectiveness demonstration program in seven Asian countries and one Africa country (Table 1). The DOMI program was conceived and funded in 1999 aiming at accelerating the introduction of existing vaccines and developing new vaccines to protect people against the enteric diseases, typhoid fever, cholera, and shigellosis [7].

While implementing the data management systems in those eight countries, we faced several challenges in management of the data systems at the local sites. Some of the challenges were common among those countries and others more country-specific. Based on our experience working with different groups in different research settings around the globe, we have constructed model data management practices for epidemiological studies in LDCs. In this paper, we outlined several important features of this model data management system.

## Results

### The data management organization

#### The office space

In LDCs, data management is often specific to a research project; it emerges when the project starts functioning and disappears at the end of project. Rarely, does one find a permanent data management setup in these epidemiological research settings. When the project starts collecting the data, an insufficient, non-dedicated, space is often assigned for data management activities. Since a large epidemiological study generates voluminous data flow, a non-dedicated, insufficient office space creates a big challenge for managing the data, checking the forms, ensuring safety and security of the data, and archiving the forms in an effective efficient manner to ensure quick and easy access to the forms.

**Figure 1:**
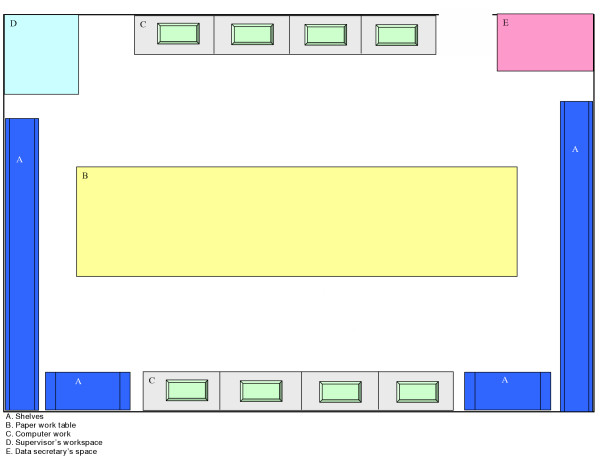
**The model data management office**. It shows all the data staff, necessary equipment, tables, file storage, should be connected.

Ideally, the data management office should have enough space to place the necessary computers, tables for paper work, locked file cabinets for safe storage of forms, and supervisor's and data workers' desks. A model data management office is shown in Figure 1. The supervisor's desk inside the data room makes him/her easily accessible to the data workers, which ensures efficient supervision and quick solutions to problems as they arise. Since data editing requires accessing of forms, keeping the file cabinets inside the data office makes the editing job easier for the data workers. Our practical experience suggests that data workers tend to edit computerized data without going through the source documents if these documents are not easily accessible (i.e. stored outside the data office).

#### The working units

**Figure 2:**
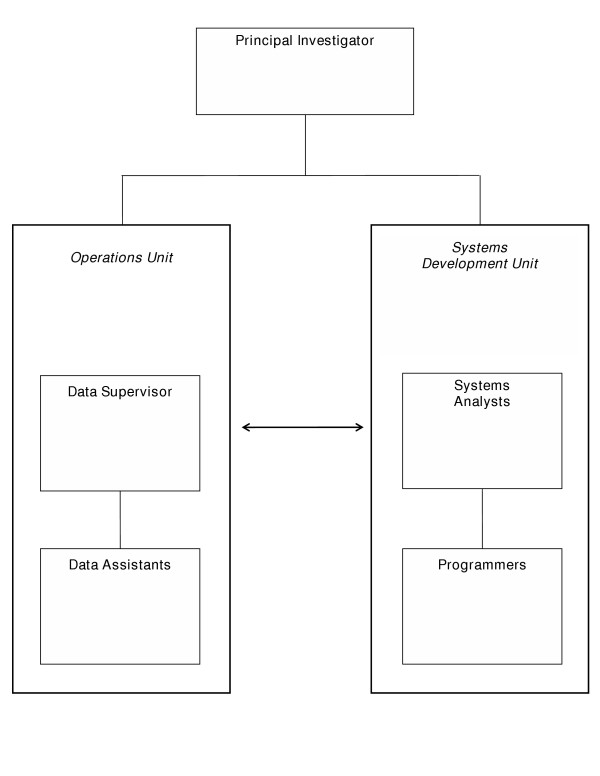
**The data management team of the Disease of the Most Impoverished (DOMI) projects**. The team included systems and operation units. The responsibilities of the team members have been described in the text.

The success of an epidemiological study depends on efficient design of the database and the performance of the systems. Investigators need to make sure the management systems reflect their needs. They are well advised to review the design before substantial amounts of time are invested in creation and implementation of the database. A model data management team is composed of two essential working units: systems development and operations. The data management team of the DOMI project is shown in Figure 2.

#### Systems development unit

The systems development unit is responsible for designing, developing, implementing, and improving the performance of data management software. Skilled professionals for designing epidemiology data systems are in short supply in industrialized countries and even more so in LDCs. The remuneration and benefits obtained from working in project-based data management of epidemiological studies in LDCs are usually insufficient to attract skilled professionals. The problem may be overcome by outsourcing the job of systems development, although there is the possibility that the outsource may not be able to respond in time of need. For the DOMI project, we have dealt with these problems by establishing the systems development unit centrally at the level of IVI. The unit consists of skilled individuals who design and develop the data systems at IVI and travel to the local sites for implementation, trouble-shooting, performance evaluation, and monitoring of the data activities.

#### Operations unit

The operations unit is responsible for the flow, entry, editing, compilation, reporting, analysis, archiving, safety and security of the data. The data staff members for the operation unit are usually recruited locally based on specific requirement criteria for the different positions.

The data supervisor is the key position of the operations unit. The supervisor has to be familiar with all the data-related activities of the project, and needs to have complete understanding of the data flow. The supervisor should be capable of resolving any technical questions arising in the data office and have good communication skills. Since the supervisor's job is essential in daily data activities, a deputy is often required to act in the absence of supervisor.

A data secretary is essential in large-scale epidemiological studies. She or he should keep track of all data forms, maintain a log of data activities, and archive the data and documents. The data operators are responsible for entering data into screens, detecting errors in the data using computer programs, and updating the erroneous data through standard protocols. Since the data operator's job is repetitive and monotonous, an honest person is preferred over a highly skilled but poorly motivated individual.

## Discussion

### Design and development of a data management system

Designing the schema of a database is an important exercise in data management. The key features of the design scheme include the number of data tables to be included in the database, normalization of the data across tables, the key fields for the data tables, and interlinking of the tables within the database. A well-designed database can reduce programming time during data analysis. In contrast, a badly designed database may delay data processing and may even make some analyses impossible.

**Figure 3:**
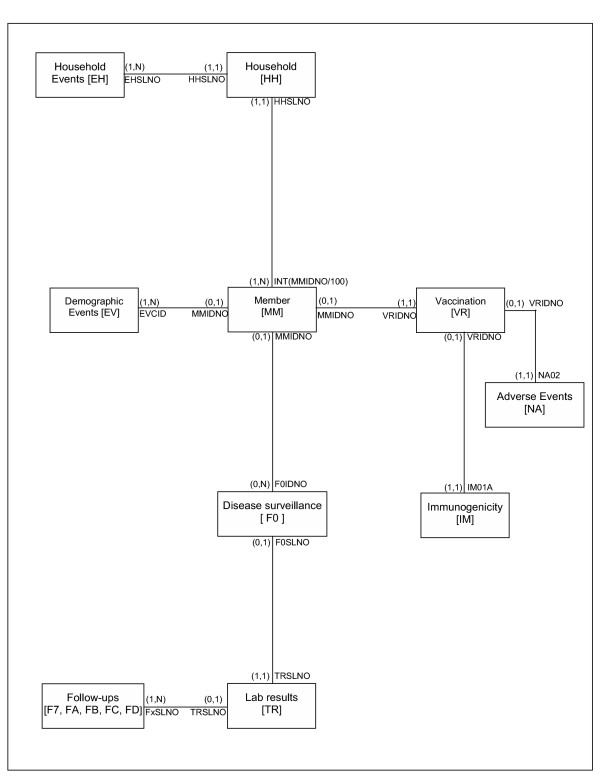
**The schema of database for the DOMI typhoid vaccine trial programs**. The data table names are shown inside brackets next to the form name. The linkage keys are shown next to the box of the data table. The logical relationships between the entities are shown in parenthesis. In a relationship (1,N), "1" indicates each entity of the table will link to at least one entity of the other table, and "N" indicates multiple entities of the table may link to at least one entity of the other table. In a relationship of (0,N), "0" indicates not all entities of the table will link to another table. And, in a relationship of (1,1), the later "1" indicates a single entity will link to at least one entity of the other table.

We prefer to use a relational database model for our epidemiological studies, as it is the most popular model among currently available data base management systems [8]. A database can be understood as a collection of related files. Early models included the hierarchical model (where files are related in a parent/child manner, with each child file having at most one parent file), and the network model (where files are related as owners and members, similar to the network model except that each member file can have more than one owner). The relational database model was a significant step forward, as it allowed files to be related by means of a common field. Any two files need to have one common field, such as an identification number, which makes the model extremely flexible. The goal of relational database design is to generate a set of schemas that allow us to store information without redundancy, and to retrieve information easily (and accurately). The schema of the relational database for the DOMI typhoid vaccine study is shown in Figure 3.

The development of a data management system for a large-scale epidemiological study can be laborious, as the data structure of such studies is usually complex [9]. An epidemiological study that generates a large numbers of observations, collected over an extended period of time, from several centers increases the data processing workload and the likelihood for discrepancies between variables. Incorporating modules for addressing issues such as error identification, data correction and processing, and data linkage may require a significant amount of development time. Additionally, a substantial amount of time may be required for testing and repairing systems.

### Implementation of data systems and training

We developed a generic data system for the DOMI epidemiological studies and field trials. The generic data system could not be replicated exactly in several countries because some of the data collection and management issues were site-specific. During the implementation phase, the generic data system was adapted at each site. Several system errors showed up during the implementation phase and required repair. Other errors became apparent during operation, and were repaired on site. Systems performance was continuously reviewed.

Practical training on the use of the data systems was given to the local data staff. Since unskilled staff were often recruited by the local site, the training program was conducted in phases. Communication was found to be a challenge in conducting the training, since the IVI staff often did not speak the local language. But through intensive use of translator and close observation during operation of the data systems, transfer of knowledge and skill could be assured.

### Hardware and software

The increasing accessibility and sophistication of computer hardware and software facilitates the use of model data management systems for health studies in resource-poor environments [10]. Note that hardware and software requirements depend on the data management needs. For the epidemiological studies in LDCs, it is safer to be conservative and use tried and tested products, especially in situations where technical backup may not be readily available [11].

Networking of computers is increasingly common and useful for optimization of organizational resources. Networking enables several machines to share the same software and printers. More than one user can simultaneously access the same database. Simultaneous use requires attention to identification of "versions". However, since our data systems allow accessing only the most recent version of the database, the operators need not require paying attention for identifying the versions while updating the database.

Our data systems are implemented in a network environment, so that multiple users can share the same database as well as other resources. A major disadvantage of networking is that if the system comes to a halt, all individuals connected may be prevented from working until the fault is repaired. This problem does not occur in a stand-alone system where each machine is running its own software systems and database. A stand-alone system can be used when several users require accessing the same data set at the same time [11]. For instance, during census data computerization, we employ additional manpower to complete the data entry within a stipulated time frame. To manage the situation, stand-alone systems are implemented by splitting the database and each portion is installed separately in several computers so that the data operators can work with the divided-up files.

Electric power supply in research sites in LDCs is subject to considerable voltage fluctuation and interruption. Thus, it is necessary to take adequate precaution against unreliable power supply. We, therefore, procured uninterrupted power supply (UPS) to prevent the loss of data and to protect the equipment.

### The management of data forms

The data secretary maintains a log of data form delivery. The data form delivery log book contains information such as number of forms delivered, when the forms were delivered, who delivered the forms, and who received the forms. The number of forms delivered to the data management is verified with the field record. Completeness of the forms is checked before data entry. Any kind of incompleteness in the data forms is brought to the attention of the field staff. To detect missing data forms, consecutive serial numbers called "form serial numbers" are marked on the form using an automatic rubber stamp. The form serial number allows easy retrieval of the forms.

The time interval between the delivery of forms and computerization of the data is kept as short as possible for two reasons: i) to reduce the chance of the data forms being lost and ii) to quickly resolve any errors of the data in the field. If the data entry process is delayed then the data forms are manually reviewed for gross omissions and inconsistencies. It is technically possible to computerize unreviewed data but this may result in a delay in the detection of errors. Long delays lower the possibility of finding respondents in the field and obtaining correct information.

The study data forms may contain both qualitative and quantitative types of variables. For the qualitative variables, either pre-coding or post-coding scheme is chosen depending on the type of variable. The pre-coding scheme is usually chosen when all possible answers are known, such as gender, marital status, etc. If the possible answers are not known (e.g. medication) then the post-coding scheme is chosen, and accordingly the data are collected in textual format. The local data staff discusses the post-coding operation with the investigators, because some post-coding requires technical knowledge in medical sciences. We recommend that post-coding be done before data computerization. Entering textual data rarely helps analysis and wastes resources.

### Data entry systems

Real-time data entry (entering the data at the time of interview in a mobile device such as a handheld computer) would be ideal. However such systems are complex to implement, and require skills beyond the current abilities of many data collectors. Furthermore direct data entry removes the source document, which is still a requirement for regulatory agencies and monitors enforcing the requirements of such agencies.

While there is great promise that such systems will facilitate data management in the future currently such an approach is experimental. Until real time data entry can be safely implemented data should be entered as soon as realistically possible to shorten the time to detection of errors and correction in the field.

**Figure 4:**
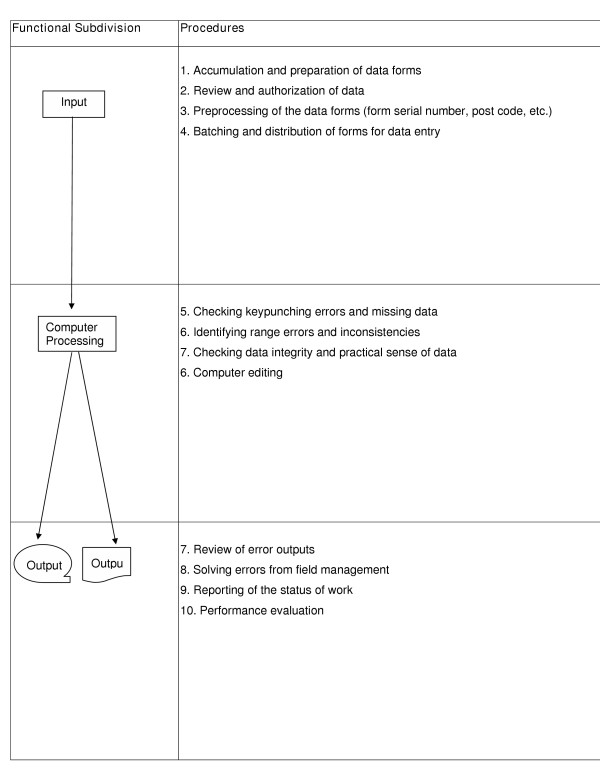
**The features of the batch processing system**. It describes operation procedures for each functional subdivision of the batch data processing system.

An interactive data entry system that detects errors while entering the data can be used but there are limitations in this system. In our experience it has been unheard of that all data forms are accurately completed in the field even in the hands of experienced, reliable operators. Interactive checks require the data entry clerk to resolve each error at the time of data entry which can slow the productivity considerably. Therefore, we implement batch processing data entry systems in our research sites. The features of our batch processing system are given in Figure 4. A batch is defined as a collection of forms within at a particular period of time. Accumulation of several deliveries of forms is also defined as a batch when the turnout of forms is low. The batch numbers correspond to generation date of the forms.

To achieve efficient and rapid data entry, the data entry screen is made to resemble the form as much as possible. The design of the data forms is also made clear and simple to achieve efficient data entry [5]. Since exploratory data analysis may not identify keypunching errors, we use double data entry systems as recommended [12]. There are two ways to design the double data entry system: single file or dual file. The former uses the original data file during 2^nd ^data entry, and identifies the error when the target entry differs from data one entered in the 1^st ^file. In this method, the 2^nd ^data entry operator is authorized to take decisions about correctness of the data. A careless or tired operator may be inclined to ignore discrepancies or may repeat the same mistake. Experience suggests that even the most efficient data entry operator commits mistakes.

Thus, we use second approach, in which the dual file system generates two separate files by two data entry operators. It is unlikely that two operators will commit the same mistake in the same data field. The two files entered by the two different operators are subsequently compared to detect keypunching errors in the entries between the two files. The dual file system allows evaluation of data entry performance (speed and accuracy) and encourages friendly competition among the data entry operators. One potential problem in the dual file system is that a clever operator may be tempted to cheat by copying and pasting the data from the 1^st ^entry. The possibility of cheating in the data entry is reduced by including a "date and time" column in the database, which is automatically updated by the system after entering a record. The supervisor routinely reviews the performance of data operators.

### Exploratory data analysis (EDA)

A double data entry system cannot detect data errors committed by the field staff during data collection. Exploratory data analysis (EDA) helps detect data errors [12]. In our systems, the EDA was set in the following six ways to identify errors in the database:

i) Sequence break finds interruptions in consecutive form serial numbers. A break in the sequence is treated as a missing form or record.

ii) Duplication detects more than one record with the same identification number, which should be unique to each record.

iii) Range error refers to the search of data that does not fall within the given choice or series. These errors are also referred to as outliers.

iv) Inconsistencies arise when the values of inter-related variables do not satisfy their relational condition. For instance, when a male respondent is recorded as pregnant. Here, the record does not satisfy the relational condition between gender and pregnancy. Both intra-record and inter-record inconsistencies are explored where appropriate. For example, if two records are related as father and son, then there must be a reasonable age difference between the two individuals.

v) Data linkage refers to the problem of unlinked entries among inter-related data tables. A correct linkage is ensured by both primary and secondary key fields. For example, in our data linkage system we match person's ID as the primary key, and gender and date of birth as the secondary keys to ensure that the linkage between the inter-related tables are correct. If any one of those key fields is not matched, then the data linkage between the inter-related tables is not established.

vi) Routine reviewing of Summary outputs (descriptive statistics of the critical variables) gives an understanding about the validity of data. The review of summary outputs may help investigators avoid gross mistakes or omissions in the data. For instance, the summary outputs of monthly patient registration by healthcare, further broken-down by age-group and gender, provides us with a useful overview of data collection.

We implement comprehensive checks in our systems for the data validation. The data check plans are reviewed by the investigators. A senior data manager runs these checks. Since the output of the checks tends to be rather extensive all data entry staff is frequently required resolving all queries in the shortest possible time.

**Figure 5:**
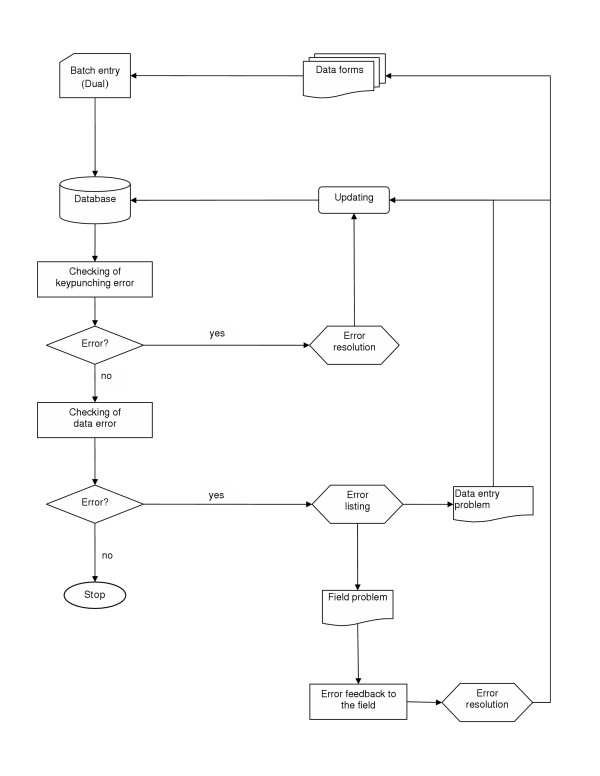
**The flow of data validation process**. The data validation process is started with dual data entry by two different persons, resolving keypunching errors, and then identifying the data errors that contain duplicate entries, outliers, inconsistencies, and data linkage problems. The data errors usually solved through field verifications unless the mistake is done at the level of data entry.

Figure 5 shows the flow of our data validation process. Since the comprehensive checks detect all kinds of errors, they create a large workload during the data validation process. However, the comprehensive checks help us to create a reliable database for our scientific research purposes.

### Resolutions of errors in the data

We classify errors into two types: data and keypunching errors. Data errors are due to incorrect data collected in the field [12,13]. Keypunching errors, which may occur during data entry, should be resolved before checking data errors. Data errors may occur in a variety of ways. In a LDC where illiteracy rate is high, subjects may not know their exact age or date of birth or even their exact name as they may be known by several names or informal nicknames [5]. Verifying a person's identification and linking his/her subsequent information to the database may therefore be challenging. In the DOMI studies, we enumerated the study population at baseline, and introduced a unique identification called census ID for our study population. A computer-based ID searching system was implemented in the clinics/hospital to locate the census ID, and to verify age and gender of the patients who presented in our target hospitals/clinics.

For all data errors, the data entry operators should not make unilateral decisions on correcting the errors. We established a standard operating procedure (protocol) for the management of data errors. According to the protocol, the data operators print the list of errors, and then check the form for possible resolution. They are allowed to correct the errors if it was a keypunching error. However, if it was not a keypunching error (i.e. the computerized data was the same as in the data form), then they report the problem to the field management for resolutions. When the corrected data are received, then the error in the database is rectified. If the correct data are not received within a scheduled time, then the field management needs to be reminded to provide immediate resolution of the problems. And, if the corrections could not be done by the field management then the error descriptions should to be documented corresponding to the data and keep them for future references.

All kinds of corrections may be reflected in the error list, data forms, and in the data files. We correct the data in both files generated by double entry, because our experiences suggest that one may introduce a new error while updating the data. Also, there is the possibility of updating the data in the wrong field. It is therefore essential to maintain an error logbook.

In paper records, applying corrector fluid over the old erroneous data is strictly forbidden. The old data are simply crossed off with a single line, and the new data recorded next to it. Against intuition, a "dirty" form is more likely to be accepted than a "clean" form. All error lists and updated documentation are kept securely for future references.

A good relationship between the data and field staff is absolutely essential. The field management should be made aware of how poor data collection can create a burden of work for the data management team. Then in turn, data management team needs to appreciate the obstacles to high-quality data collection in the field [11]. The two working groups should work in a close collaboration. Accordingly, the data management team should visit the field site to get an understanding about the problems in collecting the data and the sources of errors, and the field staff should spend some time in the data management office to participate in resolving data problems.

### Data dictionary

The data dictionary contains descriptions of data and the data fields and is one of the most essential elements of a database. Without the data dictionary, the database becomes simply a repository of meaningless numbers and characters. Our data dictionary contains the following items:

- column (variable) ID

- column description

- column type

- column length

- column length for decimal places

- minimum value of the variable

- maximum value of the variable

- other values of the variable (such as unknown, missing, etc.)

- data dictionary updated by

- date of data dictionary update

The data dictionary not only provides us a description of the data, but also facilitates us the use of generic programs to detect keypunching and data errors. The dictionary also helps us to detect any structural change in the database by the unauthorized person(s).

### Data Freezing (Locking)

Data freezing (locking) is necessary to ensure that the analysis is done based on a final version of the database. Data analysis should use exclusively the agreed upon, frozen dataset. Analysis based of earlier datasets should not be considered acceptable.

The timing of data freezing depends on the status of data set as well as field methodologies. For example, after completing the field work and cleaning the data according to the standard operating procedures, several errors may show up during the analytical stage. Some variables may be critical, and any error in those variables may change the result of analysis. Therefore, we lock a data set after careful reviewing of the critical variables. The following items are stored in a separate table in connection to the data freezing

- Database name

- Data table name

- Date of frozen

- Requested by

- Generation date of the data set

- Number of rows (records)

- Number of columns (variables)

- Size of the data set

- Frozen by

- Remarks (write the name of the link file containing unresolved problems in the data set)

### Data safety and security

Only authorized users have access to our data management systems. The data entry and editing systems store user IDs corresponding to the record entered or edited. We strongly discourage the use of common log-in information by the team members. An audit trail is implemented in the systems to keep records of the history of updates in the database. The audit trail includes data table name, identification of the record modified, column name of the table, old data, user ID, and update date.

The operations unit maintains logs of all data activities [14]. The logs of data activities are recorded in a logbook or in an electronic file. We encourage use of the electronic file, so that the status reports can be quickly produced and distributed. All kinds of data documents that include systems documents, data forms, logs of data flow, error outputs, error resolutions and the process of data cleaning are kept in a safe place for future reference. It is useful to reassure the data team that revealing many problems in the logs are not to be considered as shortcomings, but rather as an indicator of sincere work. In contrast, few or no mistakes would be suspicious.

The data forms are kept in order of form serial number, so that the retrieval of the forms is easy. Necessary measures are taken so that forms are protected from hazards such as rot, insects, and theft. All kinds of data documents are planned to be stored for at least 5 years after the project is completely over.

Anti-virus software is installed in every computer to protect the data and software against viruses. Since a database could be damaged or lost in many ways, we keep backup files, and the backups are made regularly on external storage devices. Multiple backups with at least last three generations (an update in the database creates a new generation of database) of the database are kept since errors found in a recent data set might require reviewing the previous copy of it. One backup copy is kept at the IVI, which is geographically separated location from the field sites. The logs of every backup are maintained in a logbook that contains name of the database, person backed-up, backup date, media name, and the location of the media.

Our data management systems comply with the principles that govern biomedical research involving human subjects, the Declaration of Helsinki and the Good Clinical Practice. The data management systems ensure participants' confidentiality by not allowing users to link names with the history of medical events of the study participants. Access to electronic database and hard copy data are restricted to authorized senior study personnel only.

## Conclusion

Analyses of the epidemiological studies can be flawed not only by problems in data acquisition and field methodology, but also by errors in the construction of databases. Constructing reliable databases depends on a variety of factors, not least study personnel and equipment [15,16], and practicing good data management is important. Data management in epidemiological studies should receive high priority.

We noticed a serious knowledge gap in understanding principles and practices of data management in most of our research sites in LDCs, which can be overcome by implementing the model data management system. We hope that the experience we have gathered over the years working in different LDCs has helped us to improve data management practices and may help others.

## Authors' contributions

MA suggested to summarize the experiences of the team and wrote the first draft of this paper. JKP, LVS, CJA, JLD, and JDC contributed ideas and revised sections of the paper.

## Pre-publication history

The pre-publication history for this paper can be accessed here:



## Figures and Tables

**Table 1:** Studies undertaken by the DOMI program in different countries (X indicates study undertaken)

Countries	Epidemiology			Health economics			Behavioral sciences		
	
	DP	DB	EDC	COI	VDC	WTP	Qnt	Qul	Rpd
Pakistan	X	X	X	X	X	X	X	X	X
India	X	X	X	X	X	X			X
Bangladesh		X		X		X	X	X	
Thailand		X	X	X			X	X	
Vietnam	X	X	X	X	X	X	X	X	X
China	X	X	X	X	X	X	X	X	X
Indonesia	X	X	X	X	X	X	X	X	X
Mozambique	X	X		X			X	X	

DP = Vaccine effectiveness demonstration projects; DB = Prospective disease burden studies; EDC = Systematic collection of existing data; COI = Cost-of-illness studies; VDC = Vaccine delivery cost studies; WTP = willingness to pay, studies of the private demand for vaccines; Qul = Qualitative behavioral studies; Qnt = Quantitative behavioral studies; Rpd = Rapid assessment
